# Prospective Evaluation of Cervical Scrapings CDO1 and CELF4 Methylation (epiHERA^®^) Assay in Detection of Endometrial Cancer

**DOI:** 10.3390/cancers17183010

**Published:** 2025-09-15

**Authors:** Ho-Sze Jacqueline Lee, Shiye Wu, Suet-Ying Yeung, Chun-Wai Cheung, Wen-Ying Linda Fung, Pui-Kei Sonia Kwok, Kar-Kei Yung, Tsz-Kei Sani Wong, Abhiram Kanneganti, Tat-San Lau

**Affiliations:** 1 Department of Obstetrics and Gynaecology, The Chinese University of Hong Kong, Hong Kong, China; zoeysywu@cuhk.edu.hk (S.W.); carolyeung@cuhk.edu.hk (S.-Y.Y.); kkyung@cuhk.edu.hk (K.-K.Y.); lautatsan@cuhk.edu.hk (T.-S.L.); 2 Department of Obstetrics and Gynaecology, National University Hospital, Singapore 119228, Singapore

**Keywords:** endometrial cancer, abnormal uterine bleeding, methylation assay, cervical scraping, cervical sample, diagnostic test, screening test

## Abstract

**Background/Objectives**: Our study evaluates the performance of the CDO1 and CELF4 methylation assay of cervical scrapings in diagnosing endometrial cancer. **Methods**: Patients referred for abnormal uterine bleeding, suspected endometrial pathology on imaging, endometrial hyperplasia, or cancer, who were indicated for endometrial sampling +/− hysteroscopy +/− hysterectomy, were recruited prospectively from the gynecology clinic of Prince of Wales Hospital, Hong Kong, from January 2023 to November 2024. Methylation assay was performed on the cervical scrapings of the participants, with the results compared to endometrium histology obtained on the same day. **Results**: Methylation assay results of 675 patients were compared to 629 outpatient endometrial assessments and 46 hysterectomy histology. The assay yields an accuracy of 97.3%, sensitivity of 84.1%, specificity of 98.8%, PPV of 89.2%, and NPV of 98.2%. The AUC is 0.92 with a Kappa coefficient of 0.85. The false-negative rate was 0.8% among the 629 endometrial assessment cases. Endometrial thickness, abnormal cervical cytology, and endometrial hyperplasia significantly affected the accuracy. Among seven false-positive cases, five had endometrial hyperplasia and two had cervical intraepithelial neoplasia. Eventually, three patients were diagnosed with endometrial cancer and one with cervical cancer 1 to 4 months later. **Conclusions**: Methylation assay of cervical scrapings is highly accurate in diagnosing endometrial cancer with a high sensitivity, specificity, and low false-negative rate. It can act as a triage to reduce invasive endometrial assessment. All false-positive cases were related to neoplastic processes in the genital tract, indicating that it may be useful for detecting cancer early, before histological change is evident.

## Simple Summary

This study evaluates the performance of the CDO1 and CELF4 methylation assay of cervical scrapings in diagnosing endometrial cancer. Cervical scrapings from patients with abnormal uterine bleeding, suspected endometrial pathology on imaging, endometrial hyperplasia, or cancer undergo a methylation assay, with the result being compared to endometrium histology. The assay yields an accuracy of 97.3%, sensitivity of 84.1%, specificity of 98.8%, PPV of 89.2%, and NPV of 98.2%. The AUC is 0.92, the Kappa coefficient is 0.85, and the false-negative rate is 0.8%. Among seven false-positive cases, five have endometrial hyperplasia and two have cervical intraepithelial neoplasia. Eventually, three patients are diagnosed with endometrial cancer and one with cervical cancer 1 to 4 months later. Methylation assay can be a triage to reduce invasive endometrial assessment. All false-positive cases are related to neoplastic processes in the genital tract, indicating that it may be useful for detecting cancer early, before histological change is evident.

## 1. Introduction

Endometrial cancer is the sixth most common cancer among women globally [1] and is the most common gynecological malignancy in developed countries [2]. Its incidence is on the rise globally, with 417,100 new cases reported in 2022 compared to 378,400 cases in 2018 [1,3]. It had risen from the 17th most common cancer in 2018 to the 15th most common cancer in 2024 [1,3]. The increase is anticipated to continue, driven by risk factors of endometrial cancer being increasingly common, such as an increase in obesity. Currently, there is no reliable screening test for endometrial cancer [4]. The main symptom is abnormal uterine bleeding, which is also a symptom of many benign gynecological conditions. In patients presenting with abnormal uterine bleeding, the most common investigation is transvaginal ultrasound, which measures the endometrial thickness [5]. The minimum thickness indicated for a diagnostic test with endometrial assessment is not defined, especially in pre-menopausal patients. In post-menopausal patients, an endometrial thickness cutoff at 3, 4, and 5 mm has a sensitivity of 97.0%, 94.1%, and 93.5%, respectively, for the detection of endometrial cancer [6]. For patients with an endometrial thickness of ≤3 mm, endometrial cancer is unlikely; however, the specificity is only 45.3%. A transvaginal ultrasound can reliably exclude cancer if the endometrial thickness is thin. For those with thick endometrial thickness, the chance of a false positive is high, and a lot of invasive tests will be performed in non-cancerous cases [6,7]. The current gold standard for endometrial cancer diagnosis is endometrial assessment with endometrial sampling or dilatation and curettage (D&C), many a time performed with hysteroscopy [5]. A meta-analysis found that endometrial sampling yielded a sensitivity of 99.6% to 100% in post-menopausal patients and 91% in pre-menopausal patients in identifying endometrial cancer when compared with D&C or hysterectomy histology [8,9]. However, the failure rate of outpatient endometrial sampling can be as high as 11% in post-menopausal patients [9]. Being an invasive investigation, endometrial assessment can lead to complications such as discomfort, infection, bleeding, and uterine perforation. Furthermore, it requires the input of clinicians and pathologists, leading to increased cost and diagnosis turnaround time. Around 70% of endometrial cancers are detected at an early stage with a good prognosis [10]. However, if the disease is diagnosed at a late stage, the 5-year survival rate is only 10% [10]. Therefore, an easily assessable, less invasive test with high sensitivity and a lower chance of failure is needed to diagnose endometrial cancer at an earlier stage and lower cost.

The use of DNA hypermethylation as a cancer screening and diagnostic tool has gained popularity recently. To date, studies have identified a plethora of potential molecular markers for endometrial cancer detection, including genetic mutations (POLE, PTEN, CTNNB1, PIK3CA, ARID1A, KRAS, and ARID5B genes) [11] and hypermethylated genes (BHLHE22, CCDC140, CDO1, CELF4, GALNTL6, ZNF334, ZNF662, ADCYAP1, ASCL2, CDH13, HS3ST2, HTR1B, MME, HAAO, HOXA9, RASSF1, ZSCAN12, and GYPC) [12,13,14,15,16]. DNA methylation contributes to the control of transcription, affecting processes such as normal development, silencing of genes, and gene imprinting [17]. Hypermethylation of genes and promoters is a major mechanism of inactivating tumor suppressor genes [14,18]. Carcinogenesis is accompanied by widespread DNA methylation changes within the cell [14]. An increasing number of studies on epigenetics have found a close association between DNA methylation and progression of cancer [19]. Many of these epigenetic changes occur early in tumorigenesis and are important early events in the development of cancers [14]. DNA methylation analysis can be used in almost any bodily fluid, allowing biomarkers present in blood, stool, urine, and other biosamples to be suitable for early cancer detection [14]. DNA methylation analysis can predict cancer versus normal tissue with more than 95% accuracy in breast, colon, liver, and lung cancer [20].

For endometrial cancer detection, various sample types, including intra-vaginal tampon [15], blood, urine, endometrial brush [21,22], uterine lavage [12,23], and cervical scraping [13,24] samples were investigated. In a study evaluating 120 endometrial cytological specimens, hypermethylated CDO1 showed a sensitivity of 86.4% and specificity of 90.8% for endometrial cancer detection [12]. Due to the anatomical continuity of the uterine cavity with the cervix, biological material from cervical scrapings represents a unique opportunity to detect cancerous cells in the upper genital tract.

Utilizing cervical scrapings as a potential genetic material, coupled with the use of DNA hypermethylation in specific genes, including CDO1, CELF4, BHLHE22, POU4F3, MAGI2, CADM1, MAL, miR124-2, ZSCAN12, and GYPC, has been investigated for endometrial cancer detection [13,16,21,24]. Huang et al. identified hypermethylated genes BHLHE22, CDO1, CELF4, and ZNF662 as potential targets for endometroid histology endometrial cancer detection using a methylomics database [13]. The methylation profiles from cervical scrapings of endometrial cancer patients (*n* = 50) and healthy controls (*n* = 56) were compared, and the results showed that hypermethylation of CDO1 alone, CELF4 alone, BHLHE22 alone, and any two positive among CDO1/CELF4/BHLHE22 had a sensitivity of 82%, 96%, 83.7%, and 91.8%, respectively, in screening for endometrial cancer in post-menopausal patients [13]. The specificity was 93.8%, 78.7%, 93.7%, and 95.5%, respectively [13]. The same group had further validated the combination of CDO1 and BHLHE22 methylation in cervical scrapings in 592 patients (494 analyzed) with abnormal uterine bleeding [5]. The methylation status of these two genes, coupled with age and BMI, yields a sensitivity of 92.5% and specificity of 73.8% in diagnosing endometrial cancer [5]. The test could detect both endometrioid and non-endometrioid endometrial cancer [5]. Another group from the United Kingdom (UK) analyzed cervicovaginal samples of 399 patients with ZSCAN12 and GYPC region methylation and found a sensitivity of 90.9% and specificity of 92.1% for endometrial cancer detection [16].

Methylation of CDO1 and CELF4 has been utilized in endometrial cancer detection in patients with postmenopausal bleeding or pre-menopausal abnormal uterine bleeding [25,26]. The sensitivity ranged from 85.7% to 87.5% and the specificity ranged from 87.6% to 95.9%. Another study included both endometrial hyperplasia and endometrial cancer and showed a sensitivity of 84.9% and specificity of 86.6% [27]. Cysteine dioxygenase type 1 (CDO1) is a key enzyme for cysteine catabolism and belongs to the mammalian non-heme Fe(II) dioxygenase family. There is a close relationship between the extent of CDO1 promoter methylation and the progression of malignancy. Overexpression of CDO1 will promote ferroptosis of cancer cells [28]. CELF (CUGBP Elav-like family) proteins are RNA-binding proteins with pleiotropic capabilities in RNA processing. They are responsible for alternative splicing, transcript editing in the nucleus, mRNA stability, and translation into the cytoplasm. They have been linked to universal alterations in cancer proliferation and invasion. Therefore, they are regarded as potential tumor suppressors and oncogenes [29]. Our study aimed to determine whether cervical scrapings stored in ThipPrep^®^, used in conjunction with a DNA methylation assay of genes CDO1 and CELF4 (epiHERA^®^), are effective in diagnosing endometrial cancer.

## 2. Materials and Methods

### 2.1. The Patients

This was a prospective, cross-sectional diagnostic test accuracy evaluation study of CDO1 and CELF4 methylation assays of cervical scraping samples in diagnosing endometrial cancer. The study was conducted in the Department of Obstetrics and Gynecology of the Prince of Wales Hospital, Hong Kong, from January 2023 to November 2024. Patients of ≥18 years of age attending the gynecology clinic for abnormal uterine bleeding, suspected endometrial pathology on imaging, endometrial hyperplasia, or untreated endometrial cancer, who were clinically indicated for endometrial sampling +/− hysteroscopy +/− hysteroscopic biopsy +/− hysterectomy, were invited to participate. Those who agreed to participate signed a consent form. Clinical data, including demographics (age, BMI, menopausal status), medical co-morbidities, and family history of cancer, were obtained. Endometrial thickness was assessed by transvaginal ultrasound or trans-abdominal ultrasound. Patients were excluded if they were pregnant, menstruating, had pelvic inflammatory disease, had used vaginal contraception or douches 48 h prior to the clinical visit, were using hormone replacement therapy, refused endometrial assessment, or refused to participate. Participating patients underwent standard procedures during their consultation, including history taking, physical examination with speculum and digital vaginal examination, and ultrasound for endometrial thickness and ovarian pathology. Depending on the clinical condition, outpatient hysteroscopy +/− hysteroscopic biopsy and cervical smear for cytology were also performed in some cases. The cervical scrapings for the DNA methylation assay were taken during the speculum examination before performing the outpatient endometrial sampling and hysteroscopy. If the patient was referred for endometrial cancer and planned for a hysterectomy, the cervical scrapings would be taken on the day of the hysterectomy. The DNA methylation assay, an epiHERA^®^ test, was performed on the cervical scraping, and the result was compared to the histology of endometrial tissue taken on the same day as the cervical scraping. All cervical scraping samples were deidentified and labeled with a corresponding identification number before being sent to the laboratory for a DNA methylation assay. The laboratory staff was blinded to the clinical information. The clinical information, endometrium histology results, and methylation assay results were combined in a datasheet by an investigator not responsible for performing the methylation assay. The endometrium histology was retrieved from the hospital electronic medical record system by the research staff. If the endometrial sampling histology was reported as insufficient, the routine clinical management was to consider re-sampling based on clinical circumstances. In general, the result is managed as benign if hysteroscopy is normal. Otherwise, if the patient is pre-menopausal, a repeat endometrial sampling is performed. If the patient is post-menopausal, the result is managed as benign if the endometrial thickness is ≤ 4 mm. The clinical condition of these patients was reviewed at the end of the study through the hospital electronic medical record system for any development of endometrial cancer. The result of cervical cytology taken on the day of performing cervical scrapings and within the previous 7 months was retrieved from the hospital electronic medical record system.

### 2.2. Cervical Scrapings Sampling Technique

1. Excess mucus was removed from the cervical os if required.
2. The central bristles of the broom of the cervical sampler were inserted into the cervical os while the broom was fully in contact with the ectocervix.
3. The sampler would be turned clockwise five times.
4. The broom of the cervical sampler was put into a storage bottle containing 20 mL of Thinprep preservation solution and gently shaken to allow cervical exfoliated cells to stay in the bottle.
5. The specimen was stored at room temperature between 15 °C and 30 °C.
6. If the collected specimen contains excess blood (specimen has a red color), it will be discarded and not used for testing.

### 2.3. Sample Processing (DNA Extraction, Bisulfite Conversion, and Quantitative Methylation-Specific PCR (qMS-PCR))

Cervical scraping samples preserved in Thinprep^®^ were processed within 7 days of collection. DNA was extracted using the Qiagen DNA extraction kit (Qiagen, CA, USA. Cat. no. 69504) according to the manufacturer’s protocol. The concentration and quality of the extracted DNA were determined using a NanoDrop 1000 spectrophotometer (Thermo Fisher Scientific, Waltham, MA, USA). DNA was stored at −20 °C before bisulfite conversion. Qualified DNA (500 ng) was then subjected to bisulfite conversion using the LIGHTNING Bisulfite Conversion kit (INEX Innovate, Singapore. REF: 21990501-24) according to the manufacturer’s instructions. The bisulfite-converted DNA (bis-DNA) was eluted in 65 μL of nuclease-free water (final concentration at 500 ng/65 ul, i.e., 7.7 ng/ul) and stored at −20 °C until qMS-PCR analysis.

The methylated-CDO1 (CDO1^meth^) and methylated-CELF4 (CELF4^meth^) were detected by qMS-PCR using the epiHERA^®^ Methylation Detection Real-Time PCR Kit (INEX Innovate, Singapore. REF: 21030501-24) on the multiplex qPCR instrument LightCycler 480 (Roche, CA, USA). Briefly, the levels of CDO1^meth^ and CELF4^meth^ were determined using the multiplex Taqman probe provided in the kit. The multiplex probe included FAM-labeled CDO1^meth^ Taqman probe, ROX-labeled methylated CELF4^meth^ Taqman probe, and the VIC-labeled non-CpG region of the glyceraldehyde-3-phosphate dehydrogenase (GAPDH) Taqman probe. GAPDH was used as an internal control to normalize the amount of input bisulfite DNA. The CDO1^meth^, CELF4^meth^, and GADPH were multiplexed to assess the methylation levels within the same reaction. Thermal cycling was initiated with a first denaturation step at 95 °C for 10 min, followed by 45 cycles of 94 °C for 15 secs, 64 °C for 5 secs, and 60 °C for 30 secs in a 20 μL reaction mix containing 4 μL bis-DNA (with a final amount of 30 ng), 4 μL multiplex probe, and 12 μL reaction mix. All reactions were performed in duplicate. The level of the CDO1^meth^ and CELF4^meth^ was determined by the difference between the two Ct values (ΔCt CDO1^meth^ = Ct CDO1^meth^ − Ct GAPDH and ΔCt CELF4^meth^ = Ct CELF4^meth^ − Ct GAPDH). A positive result of the methylation test is defined as either CDO1^meth^ (+): ΔCt CDO1^meth^ ≤ 8.4 or CELF4^meth^ (+): ΔCt CELF4^meth^ ≤ 8.8. The reaction was considered invalid if the Ct value of GAPDH was > 32.2.

### 2.4. Statistical Analysis

Sample size calculation was performed with an online survival sample size calculation tool (https://wnarifin.github.io/ssc/sssnsp.html). Based on an expected sensitivity of 85%, specificity of 90%, precision of 10%, endometrial cancer prevalence of 7.5%, confidence level of 95%, and dropout rate of 5%, a sample size of 689 samples was obtained. The data was analyzed with the software Statistical Package for Social Science Statistics Version 22. Descriptive statistics were used for baseline characteristics of patients. The sensitivity, specificity, positive predictive value, negative predictive value, and accuracy of investigations were calculated. Categorical data was analyzed with the Chi-square or Fisher’s exact test. Continuous data was analyzed with a t-test. The significance level was set at 0.05. Receiver operating characteristic curve analysis was used to obtain the area under the curve (AUC).

## 3. Results

**Figure 1:**
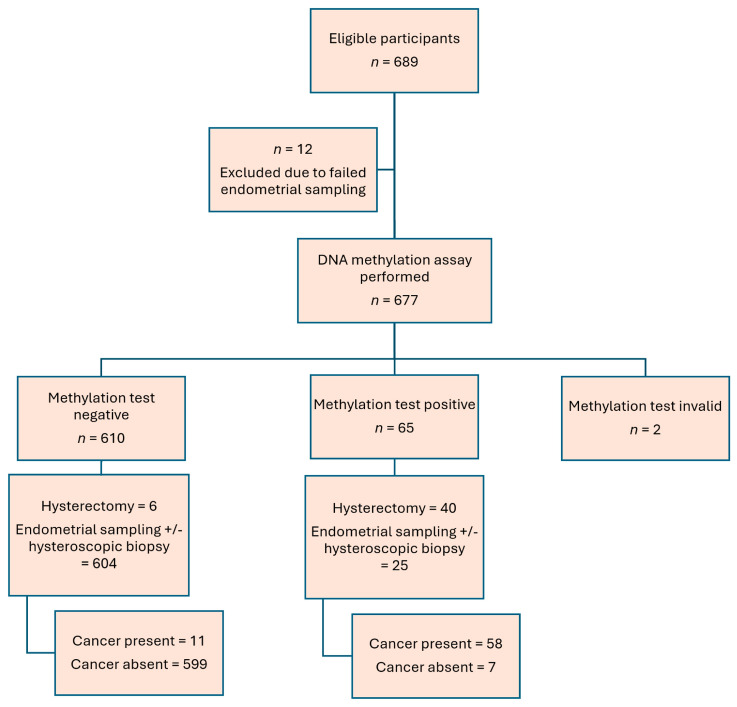
Patient recruitment flowchart.

A total of 689 cases were recruited from January 2023 to December 2024. Twelve cases were excluded due to failure to perform endometrial sampling, and two cases were excluded due to an invalid DNA methylation assay, leaving 675 cases for the final analysis (Figure 1). Among the 675 cases, 629 cases had outpatient sampling +/− hysteroscopic biopsy performed for various indications, as shown in Figure 1, and 46 cases were referred for endometrial cancer with hysterectomy performed in our unit. The demographics are shown in Table 1. All patients had cervical scrapings either on the day of outpatient endometrial assessment (*n* = 629) or hysterectomy (*n* = 46). A Pap smear for cytology was performed in 218 patients. The histology is shown in Table 1, and endometrial cancer was diagnosed in 69 patients (10.2%).

**Figure 2:**
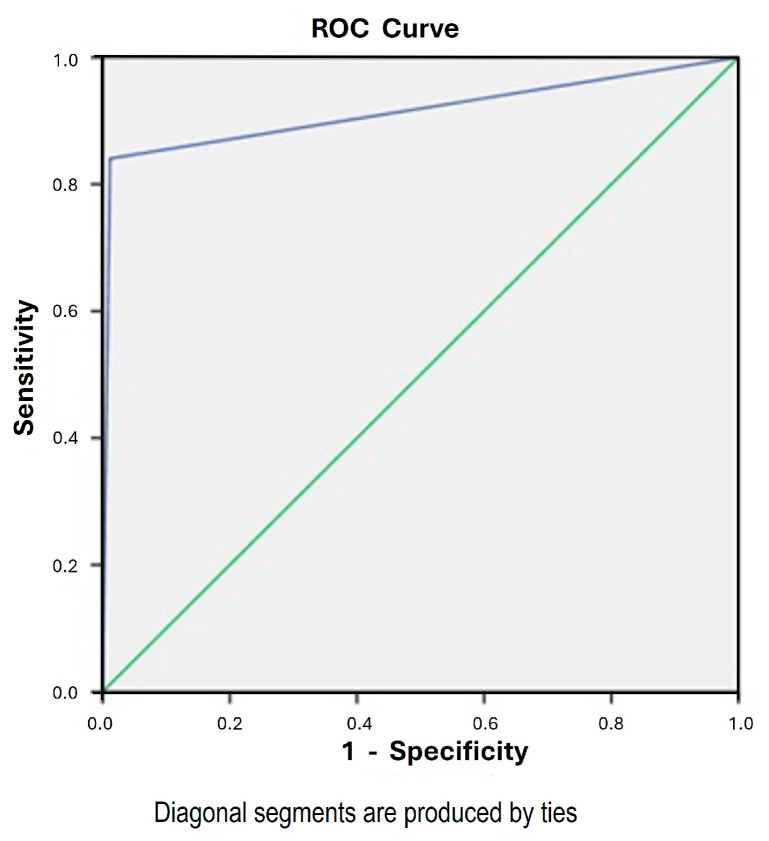
ROC curve for combined CDO1 and CELF4 DNA methylation assay in diagnosing endometrial cancer.

**Figure 3:**
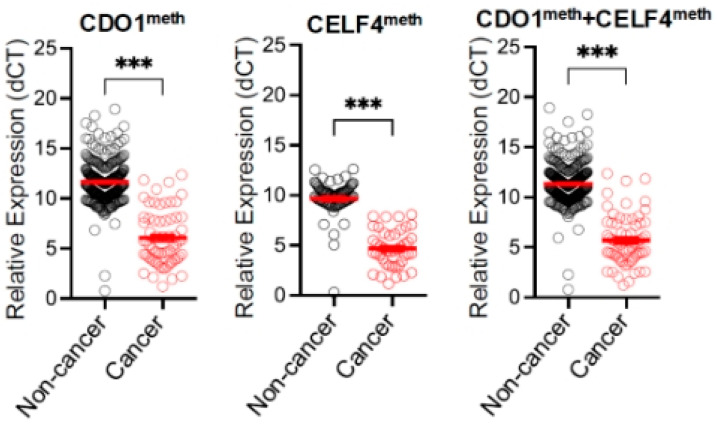
Detection of hypermethylated CDO1 and CELF4 in cervical scrapings from patients with and without endometrial cancer (EC) by quantitative methylation-specific PCR (qMS-PCR). Relative expressions normalized with GAPDH (dCT) CDO1meth [left], CELF4meth (middle), and CDO1meth + CELF4meth (right) are shown. Relative expression of CDO1meth + CELF4meth was calculated by the mean with S.E.M of dCT from CDO1meth and CELF4meth (red line). *p* values by Wilcoxon–Mann–Whitney test (*** refers to *p* values < 0.001).

The result of the CDO1 and CELF4 methylation (epiHERA^®^) assay on cervical scrapings was correlated with the endometrium histology obtained either through outpatient endometrial assessment or hysterectomy. The diagnostic accuracy of CDO1 alone and CELF4 alone in diagnosing endometrial cancer was 96.7% and 99%, respectively, with a sensitivity of 73.9% and 62.3% and a specificity of 99.3% and 98.8%, respectively. When we combined CDO1 and CELF4, taking either one positive to be a positive assay, the sensitivity was increased to 84.1%, with an accuracy of 97.3%, specificity of 98.8%, PPV of 89.2%, NPV of 98.2% (Table 2), and a Kappa coefficient of 0.85. The ROC curve is shown in Figure 2 with an area under the curve (AUC) of 0.92 (95% CI 0.86 to 0.97). The false-negative rate was 1.6% (11/675). The relative expressions of CDO1 alone, CELF4 alone, and combined CDO1/CELF4 are shown in Figure 3. In the subgroup analysis of the 629 cases with outpatient endometrial assessment, the DNA methylation assay yielded an accuracy of 98.1%, with a sensitivity of 78.3%, specificity of 98.8%, PPV of 72%, and NPV of 99.17%. The AUC is 0.89 (95% CI 0.79 to 0.99) and the Kappa coefficient is 0.74.

Endometrial thickness, the presence of high-grade or glandular abnormality on cervical cytology, and endometrial hyperplasia were found to affect the accuracy of the methylation assay (Table 3). Age, BMI, menopausal status, hypertension, diabetes, hyperlipidaemia, fibroid, adenomyosis, time interval specimen obtained from LMP, stage of cancer, histology of cancer, and grade of cancer were not found to affect the accuracy of the methylation assay (Table 3). The methylation assay performed better with a thin endometrial thickness of ≤4 mm than a thick endometrial thickness of >4 mm, showing an accuracy of 99.6% vs. 95.6%, a sensitivity of 100% vs. 84.1%, and a specificity of 99.63% vs. 98.84%. In the 282 cases with an endometrial thickness of ≤4 mm, there was only one false-positive case, which was a case of cervical intraepithelial neoplasia CIN III, and there was no false-negative case. The accuracy was lower for cases with a high-grade/glandular cervical cytology compared to low-grade or normal cervical cytology (80% vs. 98.6%). Among 10 cases with high-grade or glandular abnormality on cervical cytology, there was one false-positive case.

The accuracy was lower for cases with endometrial hyperplasia versus those without endometrial hyperplasia (54% vs. 98%). There were seven false-positive cases in our study; among them, five were cases of endometrial hyperplasia. Two cases were endometrial hyperplasia without atypia, where endometrial cancer was diagnosed in one case 4 months later. Three cases were endometrial hyperplasia with atypia, where two cases were diagnosed with endometrial cancer 1–2 months later, and one case had a fibroid. The two false-positive cases without endometrial hyperplasia had cervical preinvasive or invasive pathology, one case had ASCH on cervical cytology, and the other was found to have CIN III on endometrial sampling and was diagnosed with cervical cancer 3 months later.

Outpatient endometrial samplings were reported as insufficient in 317 cases, 301 (95%) of them being in menopausal patients and 16 (5%) being in pre-menopausal patients. These cases were taken as benign endometrial assessments, in line with the clinical management. The clinical progress of these patients was being checked 4 months after the end of the study in the hospital electronic record system, and none were found to have endometrial cancer.

## 4. Discussion

### 4.1. Performance of DNA Methylation Assay

Our study demonstrated that a combined CDO1 and CELF4 DNA methylation (epiHERA^®^) assay has a high accuracy (97.3%), sensitivity (84.1%), and specificity (98.8%) in diagnosing endometrial cancer. The AUC of the methylation assay and endometrium histology is 0.92, indicating that the assay is an excellent test for diagnosing endometrial cancer. The Kappa coefficient of 0.85 (*p* = 0.03) also indicates that the assay has a strong agreement with the endometrial assessment result and is very accurate in diagnosing endometrial cancer.

Our study results are comparable to other similar studies utilizing cervical scraping methylation assay in diagnosing endometrial cancer. Previous studies utilizing methylation assays of CDO1 and BHLHE22 coupled with age and BMI in 494 patients [5], POU4F3/MAGI2 in 30 patients [21], and ZSCAN12/GYPC in 399 patients [16] found a sensitivity of 92.5%, 83–90%, and 90.9%, respectively, and a specificity of 73.8%, 69–75%, and 92.1%, respectively, for endometrial cancer detection. Our study demonstrated that the methylation assay of two genes, CDO1 and CELF4, is also a good option amongst the many genes that have been reported for endometrial cancer diagnosis.

### 4.2. Triage for Patients at Risk of Endometrial Cancer

Based on the high sensitivity and specificity in endometrial cancer detection, the DNA methylation assay has been suggested as a triage for abnormal uterine bleeding investigation to reduce the need for invasive procedures with endometrial sampling, D&C, and hysteroscopy [5,16]. When the DNA methylation assay was applied to the Taiwan National Health Insurance dataset, a possible reduction by 69–73% was found in invasive procedures [5]. A UK study also demonstrated that the methylation assay outperformed ultrasound in diagnosing endometrial cancer in patients with abnormal uterine bleeding, advocating methylation assay to be incorporated into the diagnostic pathway to reduce the number of histological assessments with a false-negative rate of only 0.3% [16]. If the DNA methylation assay is incorporated into our cohort for triage for invasive endometrial assessment, among the 629 outpatient endometrial samplings performed for various clinical indications (Table 1), 25 methylation assays would test positive (18 true positive, 7 false positive), requiring further invasive investigations. A reduction of 96% of invasive tests (604/629) can be achieved with a false-negative rate of 0.8% (5/629).

DNA methylation assay has the advantage of being highly reproducible and less operator-dependent than transvaginal ultrasound [5], does not require any special handling of tumor specimens, and can be applied to fresh-frozen and formalin-fixed paraffin-embedded tissues with similar efficiency [14]. However, TVS can generate an immediate report, while the DNA methylation assay has a turnaround time of 1 week in our research setting, similar to endometrial sampling. The DNA methylation assay can be performed in a primary care setting without the need for specialized staff or hospitalization for invasive endometrial assessment and pathology. The risk of invasive procedures such as uterine perforation can also be minimized. In our study, only 0.3% (2/675) of cases had an invalid DNA methylation assay result. A low level of invalid results (2%, 9/399) was also reported in a UK study assessing methylation of the ZSCAN12 and GYPC regions [16]. Our findings showed a promising DNA methylation assay, which can reduce the number of invasive procedures and reduce medical costs. Our results also show that a positive DNA methylation assay can provide valuable additional information since it is found to be associated with pre-invasive and invasive cervical disease and endometrial hyperplasia, which are significant medical conditions potentially leading to malignancy.

### 4.3. Implication on Endometrial Hyperplasia Management

Endometrial hyperplasia is a significant factor affecting the accuracy of the DNA methylation assay in our study (*p* < 0.01). It is known that endometrial hyperplasia can be a precursor of endometrial cancer. The risk of developing endometrial cancer from endometrial hyperplasia without atypia is around 3%, and it is 23% for endometrial hyperplasia with atypia [30]. There are 11 cases of endometrial hyperplasia in our cohort, and 6 cases are true negatives, while 5 cases are false positives. The most common pathology associated with a false-positive result is endometrial hyperplasia (5/7, 71% of false-positive cases). Three out of five false-positive cases are diagnosed with endometrial cancer 1 to 4 months after the DNA methylation assay, indicating that the methylation assay may be able to diagnose cancer prior to any histological evidence of malignancy. The eventual development of endometrial cancer in these cases may perhaps hint that the diagnosis by endometrial sampling is inaccurate, and that endometrial sampling could be less sensitive in detecting endometrial cancer. Sampling error is known to occur with endometrial sampling [8]. However, our study design will not be able to evaluate for these since not all patients underwent a hysterectomy.

A previous study found that the number of promoter-methylated loci increases in the progression from normal endometrium to simple hyperplasia to complex hyperplasia among 24 tumor suppressor genes. In addition, aberrant DNA methylation of some tumor suppressor genes was found to be evident before endometrial carcinoma diagnosis [18]. This indicates that a DNA methylation assay can diagnose endometrial cancer early, even before a formal histological diagnosis can be made, since the genetic changes may precede the histological changes. It can be applied as a further investigation for endometrial hyperplasia to aid decision making. The management of endometrial cancer is more complex than endometrial hyperplasia since more imaging is required, and a gyne-oncologist's input is preferred since lymphadenectomy may be required. Also, endometrial hyperplasia without atypia may not receive a hysterectomy, and a case of potential endometrial cancer will be missed. A positive DNA methylation assay can aid hysterectomy decision making and allow patients to be referred to appropriate gyne-oncologic care. However, a hysterectomy was not performed in all cases of endometrial hyperplasia in our study, rendering further evaluation of true negative cases impossible. It is well known that a concurrent endometrial cancer can be present in as high as 40% of cases of endometrial hyperplasia with atypia [31]. Further studies utilizing endometrium histology from hysterectomy as the gold standard can clarify the role of DNA methylation assay in endometrial hyperplasia management.

### 4.4. Detection of Cervical Pre-Invasive and Invasive Disease

The presence of high-grade/glandular abnormality on cervical cytology was found to lower the accuracy of the DNA methylation assay (80% vs. 98.6%, *p* = 0.02). A possible explanation is the ability of the DNA methylation assay to detect cervical neoplastic processes, resulting in a false-positive result. In a study with 79 cases, DNA methylation of CADM1, MAL, and miR124-2 in cervical scrapes had been shown to be present in all cervical cancer cases [24]. However, no robust data on CDO1 and CELF4 methylation have been reported in cervical cancer. In our cohort, excluding the cases with endometrial hyperplasia, there were only two false-positive cases. Among these two cases, one had ASCH, and the other was found to have CIN III on endometrial sampling and was diagnosed with cervical cancer 3 months later. This indicates that a positive DNA methylation assay can also detect cervical pre-invasive and invasive disease. Our study has shown that methylation abnormalities of cervical scrapings are highly accurate in detecting neoplastic processes of the genital tract with a very low false-positive rate. All false-positive cases in our cohort indeed had either endometrial or cervical pre-invasive disease. Four out of the seven false-positive cases were diagnosed with cancer a few months later. Our 4-month follow-up after completion of the study also cannot entirely exclude the chance of malignancy development since the development of pre-invasive disease into cancer can take more than a few months.

Our study has the largest sample size (*n* = 675) among similar studies. Being a prospective study with laboratory staff blinded to the clinical information eliminated the chance of manipulation of the result based on clinical information. The methylation assay was compared to histology from endometrial sampling, hysteroscopic biopsy, or endometrium histology from hysterectomy. The sensitivity of endometrial sampling was reported to be 99.6% to 100% in detecting endometrial cancer [8,9]. Since the sensitivity of endometrial sampling is comparable to histology from hysterectomy, the effect of utilizing two different modalities as the gold standard on the validity of the study should be minimal.

In our study, a high number of outpatient endometrial samplings were reported as insufficient (47%, 317/675). The risk of an insufficient sample was 2% to 60%, as reported in the literature [27]. This was due to the high number (79.7%) of patients being menopausal in our study. We had checked the outcome of these patients 4 months after the end of the study, and no case was found to have endometrial cancer. Our DNA methylation assay was performed in patients at risk of endometrial cancer indicated for endometrial assessment; therefore, the assay could only be interpreted as a diagnostic aid and cannot be regarded as a screening test in an asymptomatic population. Further studies are required to clarify the role of the methylation assay for screening endometrial cancer in asymptomatic patients. In our study, GAPDH was utilized as a housekeeping gene in the methylation test. Historically, studies on gene expression in gynecological tissues have conventionally employed glyceraldehyde-3-phosphate dehydrogenase (GAPDH), 18S ribosomal RNA (18S rRNA), or β-actin (ACTB) genes [32], often selected without rigorous validation. However, recent research has indicated that GAPDH, along with ACTB and 18S rRNA, may not exhibit stable expression in endometrial cancer [33]. This instability can raise concerns about the reliability of these genes for normalization, potentially leading to inaccurate interpretations of gene expression data and impacting the overall conclusions of our study.

## 5. Conclusions

The DNA methylation assay of CDO1 and CELF4 (epiHERA^®^) on cervical scrapings has high accuracy, sensitivity, and specificity in diagnosing endometrial cancer. It can act as a triage test for the investigation of patients at high risk of endometrial cancer to reduce invasive endometrial assessment and medical costs. DNA methylation assay also identifies pre-invasive endometrial and cervical disease and can possibly detect genetic changes to neoplastic processes before histological changes in cancer are evident. This may be used to guide the management of endometrial hyperplasia and CIN.

## Figures and Tables

**Table 1:** Patient characteristics.

	Number of Patients	Percentage		Number of Patients	Percentage
**Age (*n* = 675)**			**Pap smear cytology (*n* = 218)**		
- <30–39	11	1.6%	Adenocarcinoma	1	0.5%
- 40–49	106	15.7%	Abnormal glandular cell	2	1%
- 50–59	240	35.6%	AGC not otherwise specified	6	2.8%
- 60–69	234	34.7%	ASCH	1	0.5%
- 70–79	74	11%	LSIL	2	1%
- ≥80	10	1.5%	ASCUS	12	5.5%
Mean age: 58.7, Median 59			Negative for intra-epithelial neoplasia	194	89%
**BMI (*n* = 672)**			**Endometrial assessment pathology (*n* = 675)**		
- <18.5	22	3.3%	**Insufficient**	317 (menopause *n* = 301, pre-menopause *n* = 16)	47%
				203	30.1%
				70	10.4%
				5	0.7%
- 18.5–22.9	189	28.1%	**Endometrial hyperplasia**		
- 23–24.9	148	22%	- With atypia	7	1%
- 25–29.9	212	31.5%	- Without atypia	4	0.6%
- 30–34.9	83	12.4%	**Malignant**		
- ≥35	18	2.7%	Endometrioid adenocarcinoma grade 1	40	5.9%
Mean BMI: 24.8, Median 24.1			Endometrioid adenocarcinoma grade 2	12	1.8%
Missing data = 3			Endometrioid adenocarcinoma grade 3	11	1.6%
**Menopaused**	538/675	79.7%	Carcinosarcoma	3	0.4%
**Family history of gynecological cancer**	46/674	6.8%	Clear cell carcinoma	2	0.3%
Missing data = 1			SCC	1	0.1%
**Hypertension present (*n* = 675)**	205/675	30.4%	**Stage of endometrial cancer (*n* = 68)**		
**Diabetes present (*n* = 675)**	89/675	13.2%	- 1A	27	39.7%
**Hyperlipidaemia present (*n* = 675)**	92/675	13.6%	- 1B	11	16.2%
			- II	14	20.6%
**Presentation symptoms (*n* = 674)**			- IIIA	1	1.5%
- Postmenopausal bleeding	444	65.8%	- IIIB	2	2.9%
- Menorrhagia	82	12.2%	- IIIC	12	17.6%
- Irregular menstruation	25	3.7%	- IVB	1	1.5%
- Intermenstrual bleeding	21	3.1%	Missing data = 1		
- Hypermetabolic activity in endometrium on PET	1	0.1%			
- History of endometrial hyperplasia	3	0.4%			
- Asymptomatic thickened endometrial thickness or hydrometra	46	6.8%			
- Abnormal cervical smear	3	0.4%			
- Post-coital bleeding	8	1.2%			
- Foul-smelling vaginal discharge	2	0.3%			
- Others	39	5.8%			
Missing data = 1					

**Table 2:** DNA methylation assay versus endometrial assessment pathology in diagnosing endometrial cancer.

Combine CDO1 and CELF4 Methylation Assay		Negative	Positive	Accuracy	Sensitivity (95% CI)	Specificity (95% CI)	PPV (95% CI)	NPV (95% CI)	PLR (95% CI)	NLR (95% CI)	AUC (95% CI)
Endometrial assessment pathology	Pathology: benign/insufficient	N = 599	N = 7	97.3% (657/675)	84.1% (73.3% to 91.8%)	98.8% (97.6% to 99.5%)	89.2% (79.8% to 94.6%)	98.2% (96.9% to 98.9%)	72.8 (34.6 to 153.1)	0.16 (0.09 to 0.28)	0.92 (0.86 to 0.97)
	Pathology: malignant	N = 11	N = 58								

PPV: positive predictive value, NPV: negative predictive value, PLR: positive likelihood ratio, NLR: negative likelihood ratio, AUC: area under the curve

**Table 3:** Factors affecting DNA methylation assay in diagnosing endometrial cancer.

	Accurate	Inaccurate	*p* Value
**Age (*n* = 675) (Mean)**	58.7 (SD 9.53)	59.3 (SD 10.1)	0.78
**BMI (*n* = 672) (Mean)**	24.8 (SD 4.45)	25.5 (SD 4.98)	0.51
**Menopausal status (*n* = 675)**			
Menopause	525 (97.6%)	13 (2.4%)	0.38
Not menopause	132 (96.4%)	5 (3.6%)	
**Hypertension (*n* = 675)**			
Present	200 (97.6%)	5 (2.4%)	1.0
Absent	457 (97.2%)	13 (2.8%)	
**Diabetes (*n* = 675)**			
- Present	85 (95.5%)	4 (4.5%)	0.28
- Absent	572 (97.6%)	14 (2.4%)	
**Hyperlipidaemia (*n* = 675)**			
Present	91 (98.9%)	1 (1.1%)	0.49
Absent	566 (97.1%)	17 (2.9%)	
**Fibroid (*n* = 674)**			
- Present	152 (97.4%)	4 (2.6%)	1.0
- Absent	505 (97.5%)	13 (2.5%)	
**Adenomyosis (*n* = 674)**			
- Present	10 (100%)	0 (0%)	1.0
- Absent	647 (97.4%)	17 (2.6%)	
**Time from LMP (*n* = 122)**			
- ≤ 14 days	40 (97.6%)	1 (2.4%)	1.0
- > 14 days	80 (98.8%)	1 (1.2%)	
**Endometrial thickness (*n* = 533)**			
- ≤ 4 mm	281 (99.6%)	1 (0.4%)	<0.001 *
- > 4 mm	240 (95.6%)	11 (4.4%)	
**High-grade/Glandular abnormality smear (*n* = 218)**			
- Yes	8 (80%)	2 (20%)	0.02 *
- No	205 (98.6%)	3 (1.4%)	
**Endometrial hyperplasia (*n* = 675)**			
- Present	6 (54.5%)	5 (45%)	<0.001 *
- Absent	651 (98%)	13 (92%)	
**Stage of tumor (*n* = 68)**			
- Stage 1	31 (79.5%)	8 (20.5%)	0.33
- > Stage 1	27 (90%)	3 (10%)	
**Histology of tumor (*n* = 69)**			
- Endometrioid	53 (84.1%)	10 (15.9%)	1.0
- Non-endometrioid	5 (83.3%)	1 (16.7%)	
**Grade of tumor (*n* = 63)**			
- Grade 1	32 (82.1%)	7 (17.9%)	0.18
- Grade 2	12 (100%)	0 (0%)	
- Grade 3	8 (72.7%)	3 (27.3%)	

* Statistically significant

## Data Availability

Data supporting the reported results are not publicly available.

## Abbreviations

The following abbreviations are used in this manuscript:

PPV	Positive predictive value
NPV	Negative predictive value
AUC	Area under the curve
D&C	Dilatation and curettage
BMI	Body mass index
PCR	Polymerase chain reaction
CIN	Cervical intraepithelial neoplasia
